# Improved numerical stability for the bounded integer model

**DOI:** 10.1007/s10928-020-09727-8

**Published:** 2020-11-26

**Authors:** Sebastian Ueckert, Mats O. Karlsson

**Affiliations:** Department of Pharmacy, Uppsala University, Uppsala, Sweden

**Keywords:** NONMEM, Composite score, Numeric stability

## Abstract

This article highlights some numerical challenges when implementing the bounded integer model for composite score modeling and suggests an improved implementation. The improvement is based on an approximation of the logarithm of the error function. After presenting the derivation of the improved implementation, the article compares the performance of the algorithm to a naive implementation of the log-likelihood using both simulations and a real data example. In the simulation setting, the improved algorithm yielded more precise and less biased parameter estimates when the within-subject variability was small and estimation was performed using the Laplace algorithm. The estimation results did not differ between implementations when the SAEM algorithm was used. For the real data example, bootstrap results differed between implementations with the improved implementation producing identical or better objective function values. Based on the findings in this article, the improved implementation is suggested as the new default log-likelihood implementation for the bounded integer model.

**Electronic supplementary material:**

The online version of this article (10.1007/s10928-020-09727-8) contains supplementary material, which is available to authorized users.

## Introduction

Composite scores are an outcome type of importance in many clinical trials. The observations are discrete numbers between a minimum and a maximum value. Early pharmacometric models often ignored these constraints and assumed a normal distribution for the residual error. In recent years, however, more sophisticated approaches have been proposed that respect the discrete, bounded nature of the data [1, 2]. An example of such a model is the bounded integer (BI) model that we recently proposed [3]. Conceptually, the BI model assumes a latent grid defined by quantiles of the normal distribution. Each subject’s location in that latent grid is described both in terms of its mean and variance over time. The BI model is conceptually straight forward and flexible. Previously, we showed the advantages in data-description of the BI model over a continuous variable model. In this work, the numeric stability of the implementation of the BI log-likelihood function will be the focus.

Numerical analysis is the field in applied mathematics that studies algorithms for solving the problems of continuous mathematics [4]. It is a common misconception to think that a solution for a mathematical model can be obtained by providing a computer with a formula and some numbers and that the machine will simply use those numbers to compute every term in the formula. In reality, many sophisticated algorithms are involved when computing predictions, even for simple statistical models. One of the challenges encountered when performing numerical computations revolve around floating-point arithmetic. Computers represent real numbers as a fixed number of significant digits and a fixed-length exponent [5]. Consequently, the relative accuracy for real numbers is limited, and a smallest and largest absolute value that can be represented exists. The impact of numerical underflow or overflow on pharmacomtric models is rarely discussed but might be relevant in some cases. Especially when the log-likelihood expression is explicitly implemented by the modeler such as for the BI model.

Therefore, the aim of this work is to compare two implementations of the log-likelihood of the BI model in terms of numeric stability and to evaluate the impact when using the model. The first implementation is a direct rendering of the mathematical definition for the BI model, and we will, hence, refer to it as the naive implementation. The second implementation aims at avoiding the problems of overflow and underflow, and we will refer to it as the improved implementation.

## Methods

### Bounded integer model

Let $Y_{ij}$ denote the observed score ($Y_{ij} \in \{0,\ldots ,S\}$) for subject $i$ ($i \in \{1,\ldots ,N\}$) at visit $j$ ($j \in \{1,\ldots ,M\}$). Using the inverse normal cumulative distribution function $\varPhi^{-1}$, we can define a latent grid of cut-points according to

1 $$\begin{aligned} q_s = \varPhi^{-1}\left( \frac{s}{S+1}\right) . \end{aligned}$$

The BI model defines the probability of observing a score of $s$ as

2 $$\begin{aligned} P(Y_{ij} = s) = \varPhi \left( \frac{q_{s+1}-f(\cdot )}{g(\cdot )}\right) -\varPhi \left( \frac{q_{s}-f(\cdot )}{g(\cdot )}\right) \end{aligned}$$

where $f(\cdot )$ is a general function describing the mean location of subject $i$ on the latent grid and $g(\cdot )$ is a function describing its standard deviation. One should note that due to $\varPhi (-\infty )=0$ and $\varPhi (\infty )=1$ this definition implies correct behavior at the boundaries ($s=0$ and $s=S$). In a general pharmacometric model, $f$ and, potentially, $g$ will depend on subject specific random effects, time, treatment, covariates, etc.

### Log-likelihood implementation

For the sake of simplicity, we consider in this section only one observation per subject and a simple mean variance model on the latent grid for each subject, i.e.,

3 $$\begin{aligned} f(\cdot )=\eta_i \quad {\text{and}} \quad g(\cdot )=\sigma . \end{aligned}$$

However, the derivations made directly generalize to more complex models.

#### Naive implementation

Combining Eqs. 2 and 3 yields the following straight forward expression for the log-likelihood

4 $$\begin{aligned} \log {\mathcal{L}}(\eta_i, \sigma |s) = \log \left( \varPhi \left( \frac{q_{s+1}-\eta_i}{\sigma }\right) - \varPhi \left( \frac{q_s-\eta_i}{\sigma }\right) \right) \end{aligned}$$

This expression can be implemented in any NLME modelling software that allows to specify the log-likelihood.

#### Improved implementation

For a simplified notation we define the z-scores

5 $$\begin{aligned} z_s = \frac{q_s-\eta_i}{\sigma } \quad {\text{and}} \quad z_{s+1} = \frac{q_{s+1}-\eta_i}{\sigma } \end{aligned}$$

The numerically improved implementation is the result of the three tweaks to Eq. 4.

First, due to the symmetry of the normal distribution, the following identity holds

6 $$\begin{aligned} \varPhi (z_{s+1}) - \varPhi (z_{s}) = \varPhi (-z_{s})-\varPhi (-z_{s+1}). \end{aligned}$$

This can be exploited to avoid rounding errors due to calculations of tail probabilities too close to 1 by choosing either the left-hand or right-hand side of this equation. When $z_{s}>0$ and $z_{s+1}>0$ we will use the right-hand side of the equation, otherwise the left-hand side.

For the second tweak, rather than first calculating the probabilities $\varPhi (z_s)$, $\varPhi (z_{s+1})$, substracting them, and finally taking the logarithm, we aim at working with the log-probabilities directly. We define

7 $$\begin{aligned} l_\varPhi (z) = \log (\varPhi (z)) \end{aligned}$$

and use the identity

8 $$\begin{aligned} \log (a-b) = \log a + \log (1 - \exp (\log b - \log a)) \end{aligned}$$

to rewrite the log-likelihood in Eq. 4 as

9 $$\begin{aligned}  \log {\mathcal{L}}(\eta_i, \sigma |s)&= l^+_\varPhi (z) + \log (1 - \exp (l^-_\varPhi (z) - l^+_\varPhi (z))) \\ l^+_\varPhi (z)&= \max (l_\varPhi (z_s), l_\varPhi (z_{s+1}))\\ l^-_\varPhi (z)&= \min (l_\varPhi (z_s), l_\varPhi (z_{s+1})). \end{aligned} $$

The log-probabilities can be represented with higher numerical accuracy. Furthermore, Eq. 9 is written such that $\exp (l^-_\varPhi (z) - l^+_\varPhi (z))$ avoids numerically overflow by only calculating the exponential for exponents less than 0. For this equation to be even more numerically stable, $\log (1-x)$ should be implemented using a special function that remains precise for $x\ll 1$ as suggested by Goldberg [6].

The third numerical tweak concerns the calculation of the logarithm of the cumulative normal distribution function $\log (\varPhi (z))$. Some software tools, such R, can calculate this quantity without rounding errors even for very small values of z. For other tools, such as NONMEM, we need to use an approximation. We first note that the cumulative distribution function for the normal distribution is given by

10 $$\begin{aligned} {\displaystyle \varPhi (x)={\frac{1}{2}}\left[ 1+{\text{erf}} \left( {\frac{x}{\sqrt{2}}}\right) \right] } \end{aligned}$$

where ${\text{erf}}$ is the error function. For the later, Abramowitz and Stegun in equation 7.1.13^1^ provide the bounds [7]

11 $$\begin{aligned} \frac{1}{x+\sqrt{x^2+1}} < e^{x^2} \int_x^\infty e^{-t^2} dt \le \frac{1}{x+\sqrt{x^2+\frac{4}{\pi }}} \end{aligned}$$

These bound are quite accurate for larger values of x, which is exactly when calculating $\log (\varPhi (x))$ is problematic (n.b. we can use Eq. 6 to change from $\log (\varPhi (x))$ to $\log (1-\varPhi (x))$). Re-arranging Eq. 11 and taking the logarithm yields the following bounds for the upper tail probabilities of the normal distribution:

12 $$\begin{aligned} \log \frac{\sqrt{\frac{2}{\pi }}}{\frac{z}{\sqrt{2}}+\sqrt{\frac{z^2}{2}+2}} - \frac{z^2}{2} - \log {\sqrt{2}}  \\ < \log (1 - \varPhi (z)) \le  \\ \log \frac{\sqrt{\frac{2}{\pi }}}{\frac{z}{\sqrt{2}}+\sqrt{\frac{z^2}{2}+\frac{4}{\pi }}} - \frac{z^2}{2} - \log {\sqrt{2}} \end{aligned}$$

**Fig. 1:**
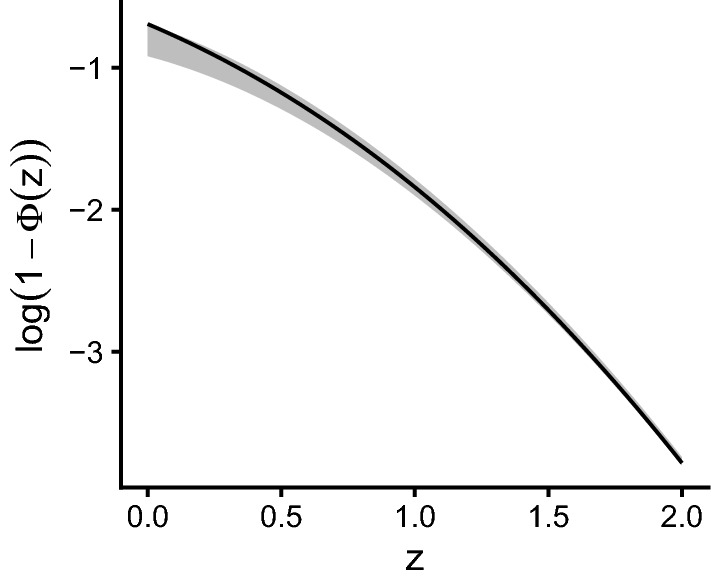
Upper and lower bound for the approximated log-probability (shown as gray area) in comparison with the exact log-probability (shown as a solid line)

Figure 1 illustrates how the precision of these bounds is increasing for increasing values of z and that already for a value of $z=2$ using either side of the inequality is an excellent approximation. When calculating the log-likelihood for the BI model, we could use the inequality to detect when the numerically calculated value of $\log (1 - \varPhi (z))$ is not within the specified bounds and then switch to use the upper or lower bound to avoid numerical problems. Alternatively, we can just use a prespecified value above which we will switch the equation. In this work, we choose the latter and will calculate

13 $$\begin{aligned} \log (1-\varPhi (z)) = {\left\{ \begin{array}{ll} \log (1-\varPhi (z)) &{}\text{ if } z \le 6 \\ \log \frac{\sqrt{\frac{2}{\pi }}}{\frac{z}{\sqrt{2}}+\sqrt{\frac{z^2}{2}+\frac{4}{\pi }}}- \frac{z^2}{2} - \log {\sqrt{2}} &{} \text{ if } z>6 \end{array}\right. }. \end{aligned}$$

### Comparison of implementations

The two implementations presented in the previous section were compared in three different contexts: (i) a more theoretical comparison by simply plotting the log-likelihood for different parameter values, (ii) a comparison within a simulation and estimation study where the simulated truth can be controlled, and (iii) a comparison using real data to assess the impact in a data analysis.

#### Graphical comparison

A graphical comparison of the naive and improved implementation of the log-likelihood expression was performed by plotting the calculated log-likelihood as a function of $\eta_i$ for the $\sigma$ values of 0.01, 0.1, 0.5 and 1. For this comparison, an observed score $s$ of 10 and a maximal score $S$ of 60 were considered.

#### Evaluation in a simulation study

The second comparison was based on a simulation and estimation study with three simulation scenarios (S1, S2, S3). All scenarios used a score with a range of 0 to 70 and the same trial design with 600 subject and 5 observations each, at times 0, 3, 6, 9, and 12 months. The function describing the evolution of the subject on the latent scale was also identical between scenarios, assuming a linear change from baseline, i.e.,

14 $$\begin{aligned} f(\beta_0, \alpha , t) = \beta + \alpha \cdot t \end{aligned}$$

**Fig. 2:**
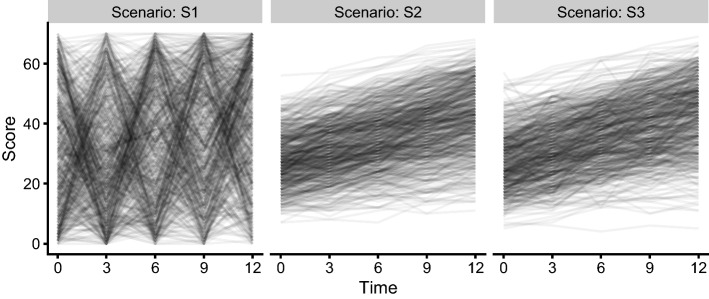
Spaghetti plot of the simulated data under each simulation scenario

The parameters $\beta$ and $\alpha$ were assumed to follow a normal distribution ($N(\theta_\beta ,\omega^2_\beta )$ and $N(\theta_\alpha ,\omega^2_\alpha )$). The model for the standard deviation on the latent scale was

15 $$\begin{aligned} g(\sigma ) = \sigma \end{aligned}$$

where parameter variability model for $\sigma$ differed between scenarios. Scenarios S1 and S2, used no between-subject variability for $\sigma$ (i.e., $\sigma =\theta_\sigma$) with typical values of 1 and 0.1, respectively. Scenario S3 used a log-normal variability model for $\sigma$ with $\log \sigma \sim N(\log \theta_\sigma , \omega_\sigma^2)$ ($\theta_\sigma =0.1$ and $\omega_\sigma^2=0.1$). The parameter values for the different simulation scenarios are summarized in Table 1. Figure 2 visualizes a simulated dataset for each of the three scenarios.

**Table 1:** Parameter values for the simulation scenarios

	Scenario		
	S1	S2	S3
$\theta_\beta$	− 0.3	− 0.3	− 0.3
$\theta_\alpha$	0.1	0.1	0.1
$\theta_\sigma$	1	0.1	0.1
$\omega_\beta^2$	1	1	1
$\omega_\alpha^2$	0.000625	0.000625	0.000625
$\omega_\sigma^2$	–	–	0.1

Each scenario was estimated using both the Laplace estimation algorithm and the stochastic approximation expectation maximization (SAEM) algorithm [8, 9]. For the later, the option ${\text{AUTO}}=1$ in NONMEM was used to let the software find the optimal estimation settings. The SSE tool in PsN was used to perform the simulation and estimation procedure [10].

The performance of the implementations for each scenario and estimation algorithm was compared in terms of the number of successful minimizations (only for Laplace), the runtime, and the relative estimation error defined as

16 $$\begin{aligned} REE = \frac{\hat{\varTheta }-\varTheta }{\varTheta } \end{aligned}$$

where $\varTheta$ and $\hat{\varTheta }$ are the true parameter value and its estimate, respectively.

#### Evaluation for real data

The third evaluation of the two implementations was performed using the data used in the work of Ito et al., originating from an observational study of patients with mild cognitive impairment or Alzheimer’s disease (AD) [11]. For the present work, only the data from the AD patients was used. The model structure and the set of included covariates was inspired by the original publication but implemented using a BI model with the following functions for $f$ and $g$ in Eq. 2:

17 $$\begin{aligned}  f(\beta_0, \alpha , t)&= \beta + \alpha \cdot t \\ g(\sigma )&= \sigma . \end{aligned} $$

The parameter model for $\beta$ the baseline, $\alpha$ the slope, and $\sigma$ the standard deviation were

18 $$\begin{aligned}  \beta&= \theta_\beta + \eta_{i,\beta } \\ \alpha&= \theta_\alpha \cdot \left( \frac{z_{i,{\text{age}}}}{75}\right)^{\theta_{\text{age}}}\cdot \theta_{\text{apoe}}^{z_{i,{\text{apoe}}}}\cdot \theta_{\text{sex}}^{z_{i,{\text{sex}}}} + \eta_{i,\alpha } \\ \sigma&= \theta_\sigma \end{aligned} $$

where $\theta_x$ denotes fixed effect parameters, $\eta_{i,x}$ denotes random effect parameters ($\eta_{i,x}\sim N(0,\omega_x^2)$), and $z_{i,x}$ denotes covariate values.

The model parameters were estimated using both likelihood implementations with the Laplace estimation algorithm in NONMEM [8, 12]. In addition, the estimation procedure was repeated for 100 replicates of the data created using the bootstrap procedure implemented in PsN [10]. Parameter estimates and objective function values (OFV), equivalent to -2 times the log-likelihood, obtained for the original data as well as the bootstrap samples were compared between implementations.

### Software

This work used NONMEM 7.4.4 for parameter estimation and simulation [12]. The simulation and estimation study as well as the bootstrap procedure were performed using PsN version 4.10.1 [10]. Data processing and generation of graphics were done using R version 3.5.2 [13].

## Results

### Graphical comparison

**Fig. 3:**
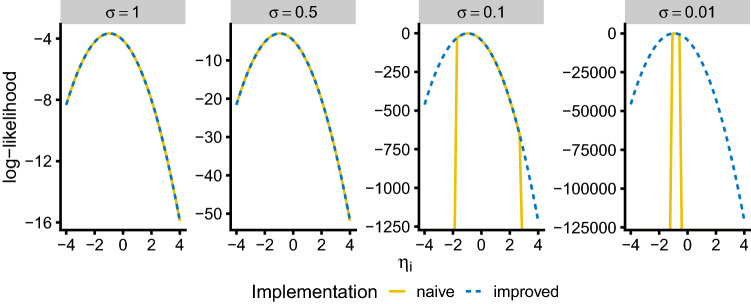
Comparison of log-likelihood implementations for different values of $\sigma$

The plots in Fig. 3 compare the two alternative implementations of the log-likelihood as a function of the $\eta_i$ parameter for different values of $\sigma$. For $\sigma$ values larger or equal to 0.5 both expressions seem to agree over the whole plotted $\eta_i$ range from − 4 to 4. For $\sigma$ values of 0.1 and smaller, however, naive and improved implementation eventually diverge significantly.

### Simulation study

**Fig. 4:**
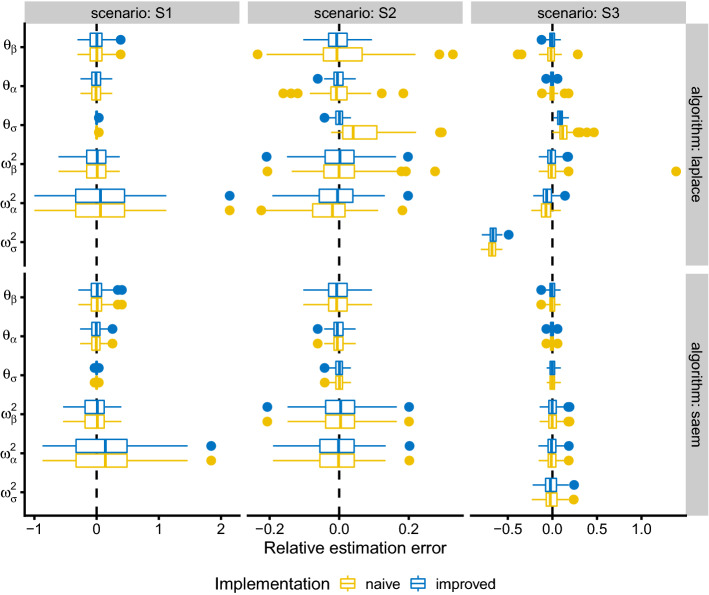
Relative estimation error for all scenarios and model parameters using the naive and improved log-likelihood implementation. The boxes show the median as well as the 25% and 75% quantiles. The lower whisker indicates the smallest observation at most 1.5 times the interquartile range away from the 25% quantile. The upper whisker indicates the largest observation at most 1.5 times away from the 75% quantile. Values outside the range indicated by the whisker is plotted as individual points

Figure 4 compares the relative estimation error obtained with the two log-likelihood implementations under the three simulation scenarios, both for the Laplace and the SAEM estimation algorithms. In addition to the relative estimation error shown in Fig. 4, the results in terms of the root mean square error (RMSE) are available in appendix B. Both implementations performed essentially identical for a $\theta_\sigma$ value of 1 (scenario S1). In scenario S2, however, the improved implementation showed significantly lower bias and imprecision when used with the Laplace estimation algorithm. The improved implementation also performed better with the Laplace estimation algorithm in scenario S3. However, the estimates for $\omega^2_\sigma$ appeared to be quite strongly biased with either implementation. There was no difference in estimation performance between implementations when using the SAEM algorithm in any scenario. Furthermore, the SAEM algorithm produced unbiased estimates even for $\omega^2_\sigma$ in scenario S3.

**Fig. 5:**
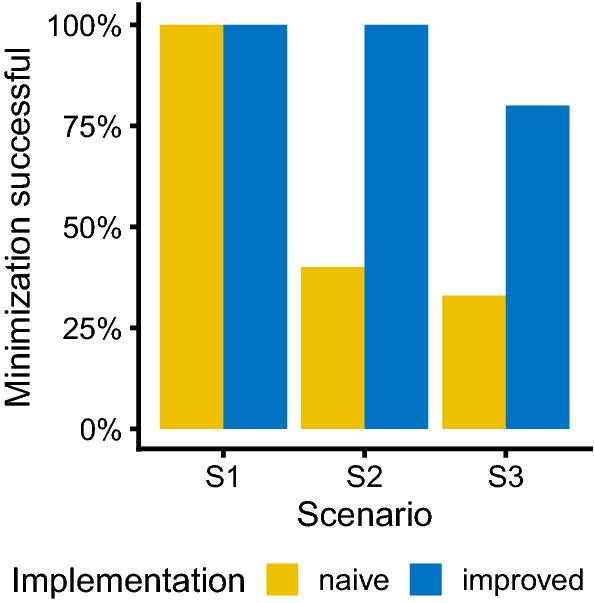
Percentage of runs completed with minimization successful under both naive and improved log-likelihood implementation when estimating using the Laplace estimation algorithm

Figure 5 highlights the differences between implementations in the percentage of runs completing with minimization successful when using the Laplace estimation algorithm. Similar to the performance in terms of relative estimation error, there are no differences for scenario S1 but quite large differences for scenarios S2 and S3. For scenario S2, the naive implementation has a success rate of less than 50% while the improved implementation maintains 100% success rate. For the last scenario, also the success rate of the improved implementation drop from 100 to 80%, but it remains considerably higher then for the naive implementation (40%).

**Fig. 6:**
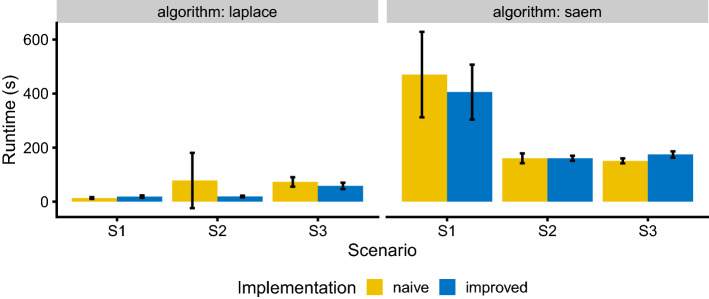
Runtime comparison of the naive and improved log-likelihood implementation for the three scenarios of the simulation study for the Laplace and SAEM estimation algorithm

The differences in runtime for the two implementations is shown in Fig. 6.

### Real data

Estimating the data from the real data example with the improved implementation yielded a 24.6 point lower OFV. The parameter estimates differed most for the covariate parameters with $\theta_{\text{age}}$ showing the largest difference (see Table 2).

**Fig. 7:**
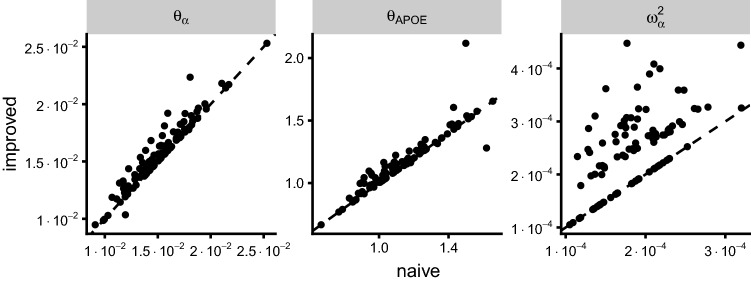
Bootstrap parameter estimates from the improved implementation plotted versus the estimates obtained with the naive implementation for a selection of three model parameters

Figure 7 conveys the impact of the log-likelihood implementation on the bootstrap results. For an arbitrary selection of model parameters, the figure contrasts the estimates for a given bootstrap samples obtained with the two implementations. While some points in the plots are located on the line of identify, indicating agreement in estimates for that particular bootstrap sample, most points are located off that line. The relative difference between estimates differed by parameter with the highest value for IIVSLOPE (60%) and the lowest for TVBASE (2%).

**Table 2:** Objective function value (OFV) and parameter estimates with relative standard error (RSE) for the real data example

	Naive	Improved
OFV	4075.1	4050.5
Estimates value (% RSE)		
$\theta_\beta$	− 0.650 (3.1)	− 0.652 ( 3.1)
$\theta_\alpha$	0.015 (17.7)	0.016 (18.8)
$\theta_{\text{age}}$	− 2.192 (30.9)	− 2.451 (29.0)
$\theta_{\text{APOE}}$	1.076 (17.0)	1.108 (18.3)
$\theta_{\text{sex}}$	1.049 (15.1)	0.987 (16.0)
$\theta_\sigma$	0.132 ( 4.0)	0.136 ( 4.0)
$\omega_\beta^2$	0.253 ( 6.1)	0.249 ( 6.2)
$\omega_\alpha^2$	0.014 ( 8.3)	0.016 ( 7.6)

**Fig. 8:**
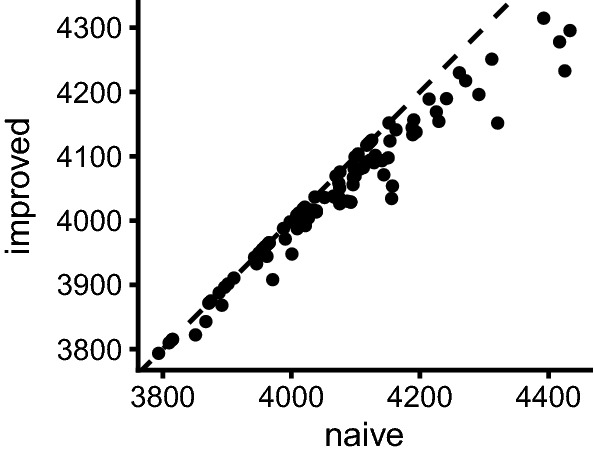
Objective function value (OFV) of the final estimates of the bootstrap samples as obtained with the naive and improved log-likelihood implementation

The impact on the OFV is highlighted in Fig. 8. The figure plots the OFVs obtained for the same bootstrap pair with the two implementations. Similar to the parameter level, some bootstrap pairs yielded identical OFV values, most, however, did not. Notable is that all points in Fig. 8 are located on or below the line of identity, indicating that the OFV obtained with the improved implementation was always equal or lower than the one obtained with the naive implementation.

## Discussion

Our results show that the improved implementation of the BI model has a considerably higher numerical stability and that this improvement can translate to meaningful differences in estimation performance. At first sight, the breakdown of the naive implementation more than 10 standard deviations away from the mean, as shown in the graphical evaluation, might appear of little practical relevance. The simulation study, however, showed that for a sufficiently small value of the variance function ($g$ in Eq. 2) these breakdowns do matter for the performance of the Laplace estimation algorithm. Finally, the real data example highlighted that small variance values could occur in a real data context.

The simulation study revealed that when the variance on the latent scale is small, the estimation properties of the Laplace estimation algorithm deteriorate considerably. The algorithm becomes more biased and imprecise as well as unstable and slow. All these negative properties increase due to difficulties in finding the maximum of the log-likelihood function for each subject. This maximization is necessary to approximate the marginal likelihood using the Laplace method. The SAEM algorithm, on the other hand, does not need to find these maxima. More importantly, the sampling-based algorithm is very well suited to handle the situation when some region of the “$\eta$-space” evaluates to a very low log-likelihood. In that case, the Markov chain will simply not explore that region and since the log-likelihood contribution from that area is low (even when considering the true log-likelihood), the estimation performance does not suffer from the numerical instability. This explains why the SAEM algorithm performs equally well with both implementations.

For scenario S3, the Laplace algorithm was not able to accurately estimate the inter-individual variability of the within-subject variability parameter $\sigma$, independent of the log-likelihood implementation. SAEM, in contrast, achieved accurate estimates with both implementations. These findings indicate that for more complex within-subject variability models the likelihood approximation performed by the Laplace algorithm might be too crude and the use of the SAEM algorithm could be indicated. In terms of runtime, the SAEM algorithm is much slower than the Laplace algorithm, even more so when the log-likelihood surface is rather flat (as in scenario S1 with a $\theta_\sigma$ value of 1).

A disadvantage of the numerically more stable log-likelihood implementation, presented here, is its increased complexity and a resulting higher risk of coding errors. In order to mitigate these risks, we have made the improved implementation available in the R package piraid which can generate scaffold code for a simple BI model [14]. The package provides an aid for the pharmacometric modelling of composite score outcomes and currently supports the BI, the coarsened grid, and the continuous variable model, as well as, item response theory models [15]. For the BI model, the user can select the desired log-likelihood implementation and we intend to make the improved implementation the default going forward.

Even if the topic of numerical stability might be particularly relevant for the BI model (due to the involvement of the cumulative normal distribution function), our findings should encourage modellers to consider numerics also for other types of models. This is especially important when implementing models using the log-likelihood expression directly. Some of the numerical tweaks used in this paper can be helpful and future research should investigate their value in other contexts.

### Electronic supplementary material

Below is the link to the electronic supplementary material.

Supplementary material 1 (mod 8 KB)

## Appendix: NONMEM control stream

The implementation of the improved model as a NONMEM control stream is sketched below. The code contains annotations, and the repetitive sections handling the various score levels have been abbreviated. A complete, runnable control steam as well as a simulated dataset from scenario S1 is provided as supplementary material.

## RMSE result from the simulation study

The following figure shows the results of the simulation study in terms of the root mean square error (RMSE) calculated as

$$\begin{aligned} {\text{RMSE}} = \sqrt{\frac{1}{M}\sum_{i=1}^M(\hat{\varTheta }-\varTheta )^2} \end{aligned}$$

where $M$ is the number of simulations performed, $\varTheta$ and $\hat{\varTheta }$ are the true parameter value and its estimate, respectively.

The results are in agreement with the plot of the relative estimation error plot shown in Fig. 4. Estimation with the Laplace estimation algorithm yields lower RMSE values when using the improved implementation in scenarios S2 and S3 but no differences between implementations for scenario S1. The SAEM algorithm produced identical results with both estimation algorithms.

## Footnotes

1 The authors learned about these bounds through the blog post “Computing extreme normal tail probabilities” by Cook (https://www.johndcook.com/blog/2018/06/01/computing-extreme-normal-tail-probabilities/).
